# Comments to Role of upper airway ultrasound in airway management

**DOI:** 10.1186/s40560-016-0204-x

**Published:** 2017-01-13

**Authors:** Wan-Ching Lien

**Affiliations:** Department of Emergency Medicine, College of Medicine, National Taiwan University and National Taiwan University Hospital, Hsin-Chu Branch, No.25, Lane 442, Sec.1, Jingguo Rd, Hsinchu City 300, Taiwan

**Keywords:** Ultrasound, Airway, Air–mucosa interface, Double-tract sign

## Abstract

Tracheal ultrasound can be an alternative diagnostic tool in airway management, besides traditional confirmatory methods such as capnography and auscultation. The standard image is a hyperechoic air–mucosa (A–M) interface with a reverberation artifact posteriorly (comet-tail artifact). If the second A–M interface appears, which we call a “double-tract sign,” esophageal intubation is considered.

With great interest, I read the article by Osman et al., entitled “Role of upper airway ultrasound (US) in airway management” [1]. The authors reviewed thoroughly for the various US applications for the upper airway, including prediction of endotracheal tube (ETT) size, difficult laryngoscopy, airway device placement and depth, percutaneous cricothyroidotomy, prediction of post-extubation stridor, and evaluation of the epiglottis.

Auscultation, waveform capnography, and chest X-ray are traditional methods for airway confirmation at critical or emergency situations. Although capnography is considered as the most reliable method, it may be biased by low cardiac output, low pulmonary flow, or epinephrine use in cardiac arrest patients [2]. US can be an alternative diagnostic tool for these conditions.

The standard image of tracheal US is a hyperechoic air–mucosa (A–M) interface with a reverberation artifact posteriorly (comet-tail artifact), surrounded by the thyroid glands. The esophagus is located at the posterior area of the left lobe of the thyroid gland [1]. Tracheal intubation is identified if only one A–M interface is present with one comet-tail artifact. If the second A–M interface appears, which we call a “double-tract sign”, esophageal intubation is diagnosed [3, 4]. However, in the session of the ETT confirmation, the authors suggested that the “double-tract” or “double-lumen” sign was present when ETT position was in the trachea. There may be some misunderstandings.

## Abbreviations

A–M: Air–mucosa interface
ETT: Endotracheal tube
US: Ultrasound
